# Eye-hand coordination during visually-guided reaching in children with monocular deprivation amblyopia^☆^

**DOI:** 10.1016/j.visres.2025.108708

**Published:** 2025-10-02

**Authors:** Krista R Kelly, Mina Nouradanesh, Reed M Jost, Christina S. Cheng-Patel, Eileen E. Birch, Serena X. Wang, James Y. Tung, Ewa Niechwiej-Szwedo

**Affiliations:** a School of Optometry and Vision Science, University of Waterloo, Waterloo, ON, Canada; b Department of Community Health Sciences, Max Rady College of Medicine, University of Manitoba, Winnipeg, MB, Canada; c Retina Foundation of the Southwest, Dallas, TX, United States; d Department of Ophthalmology, UT Southwestern Medical Center, Dallas, TX, United States; e Department of Mechanical and Mechatronics Engineering, University of Waterloo, Waterloo, ON, Canada; f Department of Kinesiology and Health Sciences, University of Waterloo, Waterloo, ON, Canada

**Keywords:** Monocular deprivation amblyopia, Unilateral cataract, Eye-hand coordination, Reaching, Ocular motor dysfunction

## Abstract

Monocular deprivation (MD) amblyopia caused by a dense unilateral congenital or infantile cataract leads to both sensory and ocular motor deficits, which can in turn affect motor performance. Previous research shows reduced fine motor skills in children with MD amblyopia on standardized tasks. Here, we evaluate eye-hand coordination during visually-guided reaching in MD amblyopia. A group of 17 children aged 7–15 years with MD amblyopia resulting from a unilateral cataract and a group of 41 age-similar control children were enrolled. During binocular viewing, children’s reaching movements (LEAP Motion Controller) and eye movements (EyeLink 1000 binocular eye tracker) were recorded as they reached to touch a dot displayed at one of four locations (±5 deg or ±10 deg) on a computer monitor. Saccade and reach kinematic measures were assessed between groups, and factors associated with impairments in the MD amblyopia group were evaluated. The MD amblyopia group as a whole had impaired saccade (lower saccade gain, reduced saccade precision, more reach-related saccades) and reach (longer total reach duration, slower peak velocity, reduced touch accuracy) kinematics compared to controls. However, performance was worse in those with a poorer visual acuity outcome (≥0.7 logMAR) compared to good visual acuity outcome (≤0.6 logMAR). MD amblyopia impacts the development of eye-hand coordination during reaching, particularly in those with a poorer visual acuity outcome. Longer deceleration in the final approach and more reach-related saccades may suggest an inability to adapt or form an efficient compensatory strategy and may also be indicative of impaired on-line control.

## 1. Introduction

Monocular deprivation (MD) amblyopia results when the eye’s ability to form a clear image is disrupted during a critical period of visual development early in life, for example from a unilateral cataract. The combined effects of stimulus deprivation and interocular suppression of discordant visual input result in severe visual deficits. Even when the duration of deprivation from a unilateral cataract is reduced with early cataract extraction and optical correction, vision can be impacted. This deprivation typically results not only in decreased affected eye visual acuity (i.e., amblyopia), but also abnormal or absent stereoacuity, interocular suppression, motion perception deficits, and ocular motor dysfunction (Birch et al., 2012; Birch et al., 1998; Birch et al., 1993; Birch and Stager, 1996; Giaschi et al., 2024; Felius et al., 2014). These deficits have the potential to disrupt the development of visuomotor skills and eye-hand coordination. Previous studies have reported impaired fine motor skills on standardized tests of motor ability during binocular viewing in children with MD amblyopia, with deficits in manual dexterity, aiming, and catching (Celano et al., 2015; Kelly et al., 2020; Webber et al., 2008). Further, poorer visual acuity outcomes were associated with lower motor performance.

Effective reaching and object manipulation rely on precise coordination of the eyes and hands. Binocular vision provides crucial depth cues (i.e., ocular vergence for absolute depth and binocular disparity for relative depth) that support our interaction with objects in three- dimensional (3D) space. These cues allow us to determine object location, contribute to planning our movements, and guide the arm during reaching (Melmoth and Grant, 2006; Melmoth et al., 2007; Niechwiej-Szwedo et al., 1869). Reaching involves an acceleration phase reflecting feedforward control (i.e., motor planning) and a deceleration phase reflecting online feedback control (Hay, 1979). Balanced binocular visual experience early in life provides vital sensory information for optimum development of eye-hand coordination (Grant et al., 2014; Niechwiej-Szwedo et al., 2020; Niechwiej-Szwedo et al., 2020). Thus, discordant binocular input in the earlier years may interrupt this development. Young children aged 4–8 years with strabismic, anisometropic, or combined mechanism amblyopia take longer to reach in the final approach during grasping, and this deficit is associated with poor binocularity (Grant et al., 2014; Suttle et al., 2011). Recently, we reported slow visually-guided reaching, particularly in the final approach (i.e., deceleration phase) in older children with strabismus aged 7–12 years, with poor binocular function being related to performance (Kelly et al., 2021). Ocular motor dysfunction accompanied the slow reaching, which included slower saccade-onset, reduced saccade precision (i.e. increased variability in gain between trials), and increased frequency of reach-related corrective saccades (Kelly et al., 2022). Together these data suggest an inability to adapt or form an efficient compensatory strategy during reaching or point to an impairment of online-control. Similar to strabismus, MD amblyopia results in severe binocular disruption; thus, like strabismus, MD amblyopia may also impact eye-hand coordination during visually-guided reaching.

Here, we investigated eye-hand coordination under natural binocular viewing conditions in children with MD amblyopia as they performed a task that required reaching out and touching a dot on the screen. Our aim was to assess how MD amblyopia affects the development of visually-guided reaching in children and to examine factors linked to potential motor deficits, such as visual acuity outcome and age of onset. Similar to children with strabismus (Kelly et al., 2021; Kelly et al., 2022), we hypothesized that children with MD amblyopia will be slower to saccade to and to reach to the target than controls. In addition, we predict they will generate more reach-related saccades than controls. These data will add to this research area, contributing to our understanding of the effects of form deprivation on eye-hand coordination.

## 2. Methods

### 2.1. Participants

A group of 17 children (mean age ± standard deviation [SD] = 10.4 ± 2.9 years, range = 7.1–15.0 years, 11 female) with MD amblyopia were enrolled following extraction of a dense unilateral congenital or infantile cataract. Congenital cataracts were dense nuclear cataracts associated with mild microphthalmia diagnosed < 6 months of age. Infantile cataracts were anterior subcapsular, nuclear sclerosis, or posterior lenticonus/posterior subcapsular cataracts diagnosed between 6 months to 28 months of age. Diagnosis, cataract extraction date, current eye alignment, and treatment history were obtained from medical records provided by the child’s referring ophthalmologist. We also enrolled 41 age-matched control children (10.4 ± 2.4 years, range = 7.0–15.7 years, 20 female) with age-typical visual acuity and stereoacuity and no history of visual disorders. Data from 32 of the 41 control children were previously reported (Kelly et al., 2022). Testing was conducted with habitual optical correction, verified through medical records. Exclusion criteria included preterm birth (<37 weeks gestation), co–existing systemic or ocular conditions, congenital infections or malformations, (neuro)developmental delay, and arm length (shoulder to fingertip) under 50 cm. All children had English as their primary language.

### 2.2. Ethics

The study was approved by the University of Texas Southwestern Medical Center Institutional Review Board, and complied with the requirements of the United States Health Insurance Portability and Privacy Act and the Declaration of Helsinki principles. Before testing began, parents (legal guardians) provided informed consent. Assent was obtained from children aged 10 years or older.

### 2.3. Procedure

#### 2.3.1. Vision Assessment

Crowded monocular best-corrected visual acuity was assessed using the electronic Early Treatment Diabetic Retinopathy Study (e-ETDRS) protocol (Beck et al., 2003) (Precision Vision^®^ Inc; Illinois, USA) scored in logMAR. Fusion at near (33 cm) was measured with the Worth 4-dot fusion test (Bernell^®^ Corporation; Indiana, USA) (Rosenbaum et al., 1999). Failing the Worth 4-dot at near indicates both central and peripheral suppression (i.e. extends across the visual field). Stereoacuity was assessed with the Randot Preschool Stereoacuity and Stereo Butterfly Tests (Stereo Optical, Inc; Illinois, USA) (Birch et al., 2008) and converted to log arcsec (1.3 – 3.3 log arcsec). No stereoacuity (‘nil’) was assigned 4 log arcsec.

#### 2.3.2. Visually-guided reaching

The protocol has been previously reported in children with strabismus (Kelly et al., 2021; Kelly et al., 2022). Here, children were seated in front of a table with their head stabilized in a head/chin rest. Viewing distance was 35 cm. Children were tested wearing their habitual optical correction, if needed, and viewed the display monitor binocularly. Using their self-reported dominant hand, children were asked to grasp a stick with their index finger and thumb (i.e., initial hand position). The stick was positioned at the body’s midline on the table 5 cm in front of their eyes. The Leap Motion Controller (LMC, software version 4.0; Leap Motion Inc., San Francisco, CA, US) was positioned 10 cm in front of the starting hand position to record hand movements. A 500 Hz binocular eye tracker (EyeLink 1000; SR Research, Ontario, Canada) was used to simultaneously record eye movements. The eye tracker was positioned 45 cm from the child’s eyes, placed above and behind an LCD display to prevent the hand or display from blocking the eye tracker’s view. Due to the complexity of the setup and testing, two experimenters ran the protocol – one viewed the eye movements and advanced the trials, while the other viewed the child’s reaching movements and determined touch accuracy.

During binocular viewing, separate calibrations were performed for the eyes (fixate on each dot for 4 s) and for the index finger (touch each dot as accurately as possible). The calibration target was a small 0.3 deg white dot presented from left to right in 5 separate horizontal locations (0 deg, ±5 deg, ±10 deg). Following calibration, the child fixated a red dot in the middle of a 1.4 deg white cross centered on the screen. When the cross vanished, a 0.3 deg white dot was displayed at one of four locations along the horizontal meridian (±5 deg or ± 10 deg from fixation). The child’s task was to reach and touch the dot as fast and accurately as possible with their index finger. For each trial, touch accuracy was recorded by an experimenter who observed whether the child’s finger fully covered the small 0.3 deg dot (‘touched’) or if the dot remained visible (‘missed’). The observer was thoroughly trained and their viewpoint was standardized, with every observation viewed from the same perspective. Dot locations were randomized throughout the experiment, with 10 trials per location totaling 40 trials (4 practice trials, 36 experimental trials). Testing took approximately 15 min.

#### 2.3.3. Data processing

##### Saccade Kinematics.

**Fig. 1.**
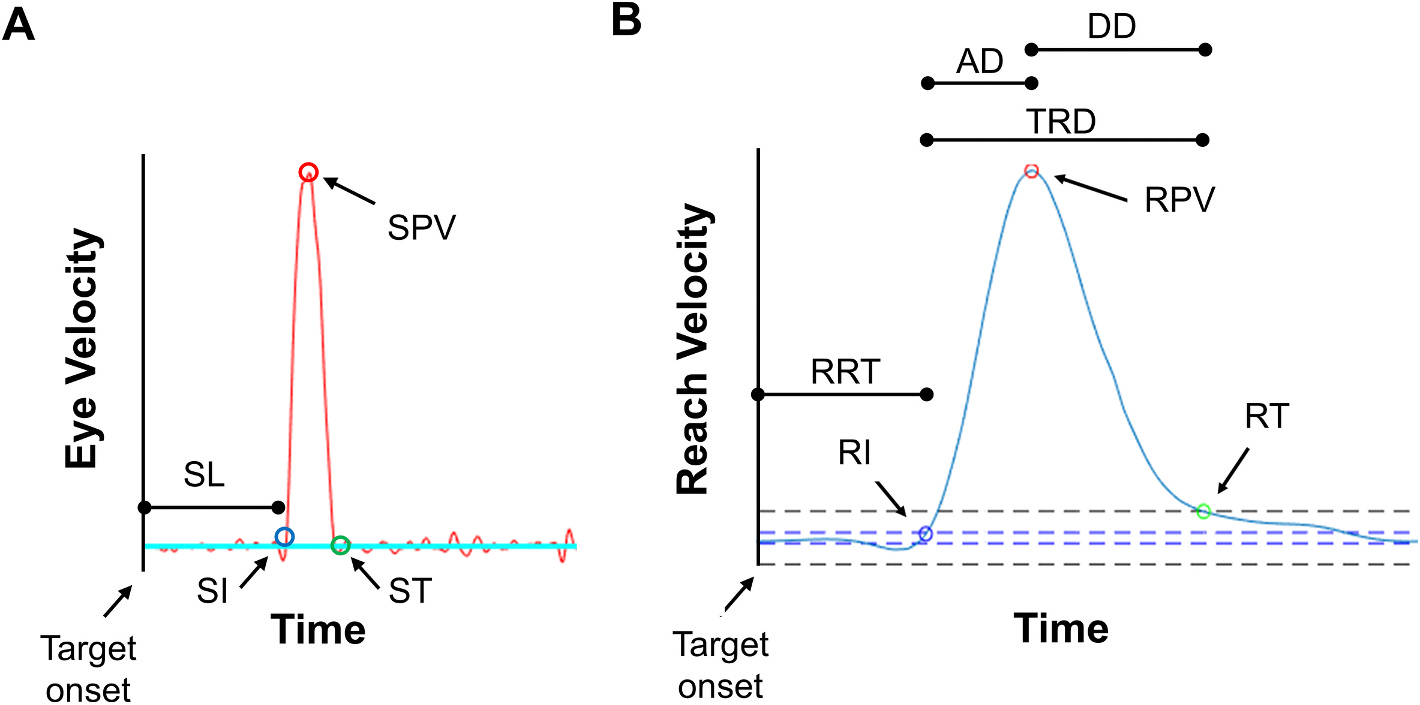
Panel A shows the velocity trajectory of the eye (red line). Saccade kinematics measures were based on velocity thresholds for saccade initiation (SI, blue circle), saccade peak velocity (SPV, red circle), and saccade termination (ST, green circle). Saccade latency (SL) was determined as the time from target onset to saccade initiation. Panel B shows the velocity trajectory of the index finger (blue line). Reach kinematics measures were based on velocity thresholds (dotted lines) for reach initiation (RI, blue circle), reach peak velocity (RPV, red circle), and reach termination (RT, green circle). From these thresholds, reach reaction time (RRT), total reach duration (TRD), acceleration duration (AD), deceleration duration (DD) were determined. (For interpretation of the references to colour in this figure legend, the reader is referred to the web version of this article.)

Eye position data for both eyes were processed using a custom-built MATLAB script (Mathworks Inc, Natick, MA, USA) previously documented (see (Kelly et al., 2022) for more information). Saccades were identified using a velocity threshold of 30 deg/sec (Fig. 1A) (Salman et al., 2006). Trials were inspected visually by a trained observer to confirm that saccades were correctly identified. A primary saccade was the first saccade that occurred within 80–1000 msec after target onset in the correct direction. The minimum time frame of >80 msec was chosen to rule out express saccades that occur within 80 msec of target onset which are anticipatory (i.e., not visually-guided) (Kingstone and Klein, 1993). The maximum time frame of 1000 msec was chosen to ensure that we did not exclude trials from amblyopic children who may be slower in their reaction time due to their eye condition. Secondary saccades that occurred within 50–250 msec after primary saccade end were considered ‘corrective’. Secondary saccades that occurred >250 msec after primary saccade end and during the reach were considered ‘reach-related’. The distinction between corrective and reach-related saccades is based on research showing that corrective saccades typically occur within 250 msec after the primary saccade ends; thus, any saccades >250 msec were not corrective and were instead considered reach-related (Cohen and Ross, 1978; Tian et al., 2013). To reduce the likelihood of misclassifying a microsaccade as a corrective or reach-related saccade, only saccades measuring ≥0.4° were included. Trials were visually inspected by a trained observer to ensure that quick phases of fusion maldevelopment nystagmus (FMNS) were not identified as corrective or reach-related saccades. The primary saccade, corrective saccade, and reach-related saccade were summed to calculate the final gain of each saccade (‘final saccade gain’). Trials were discarded if data were missing due to blinks or loss of eye tracking, or if artifacts occurred between 250 msec before target onset and the end of the primary saccade. Mean saccade kinematic outcome measures are described in Table 1.

##### Reach Kinematics.

Hand movement data were collected using the LMC with a custom-built Java application using the LMC Software Development Kit (Core Assets 4.1.1). Position data from the index finger were extracted from the LMC and analyzed with a custom-built MATLAB (Mathworks Inc, Natick, MA, USA) program that followed established signal processing techniques (Niechwiej-Szwedo et al., 2020; Niechwiej-Szwedo et al., 2018) that were based on prior validation of the LMC against the Optotrack motion capture system (Niechwiej-Szwedo et al., 2018) and have previously been published (see (Kelly et al., 2021; Kelly et al., 2022) for more information). Based on criteria in previous literature evaluating reach kinematics (Niechwiej-Szwedo et al., 2018; Gnanaseelan et al., 2014), two kinematic events were identified: reach initiation (velocity exceeding 20 mm/sec) and reach termination (velocity falling below 100 mm/sec). Using these events, we calculated mean reach kinematic measures described in Table 1, also illustrated in Fig. 1B.

##### Temporal Eye-Hand Coordination.

By combining reach kinematics with primary saccade latency, we calculated; 1) saccade-to-reach planning interval – the time available for reach planning following the end of the primary saccade, and 2) saccade-to-reach peak velocity interval – the time from when the eyes were near the target to the end of the initial phase of reach execution. Both measures are described in Table 1.

#### 2.3.4. Statistical analyses

Independent t-tests were used to evaluate differences between the MD amblyopia and control groups for each outcome measure (Table 1). To determine factors related to performance, independent t-tests were conducted to determine the effect of cataract type (congenital vs. infantile), prior strabismus surgery (yes vs. no), and affected eye visual acuity (good visual outcome vs. poor visual outcome). Based on previously used criteria (Birch et al., 1993; Taylor, 1989; Beller et al., 1981), amblyopic eye visual acuity was categorized as either a good outcome of ≤0.6 logMAR (equivalent to 20/80 or better) or a poor outcome of ≥0.7 logMAR (equivalent to 20/100 or worse). Pearson *r* correlations were conducted for the MD amblyopia group only to examine relationships of motor performance with affected eye visual acuity. Because none of the children with MD amblyopia had measurable stereoacuity and only 14/17 suppressed at near (i.e., suppression extended into the periphery; Table 2), no comparisons were conducted to determine a relationship between stereoacuity or fusion and visually-guided reaching outcomes. Mann Whitney U tests were used to analyze non-normal data based on the Shapiro-Wilk test of normality. Cohen’s *d* was used to report effect size. Holm’s sequential Bonferroni was used to correct for multiple comparisons and adjust p values. Holm’s corrects for Type I error as well as the traditional Bonferroni while maintaining greater statistical power (Eichstaedt et al., 2013).

## 3. Results

The MD amblyopia group was not different than the control group in age (p = 0.94) or arm length (p = 0.47). Table 2 provides descriptive statistics for clinical and sensory information per group.

### 3.1. Saccade kinematics

**Fig. 2.**
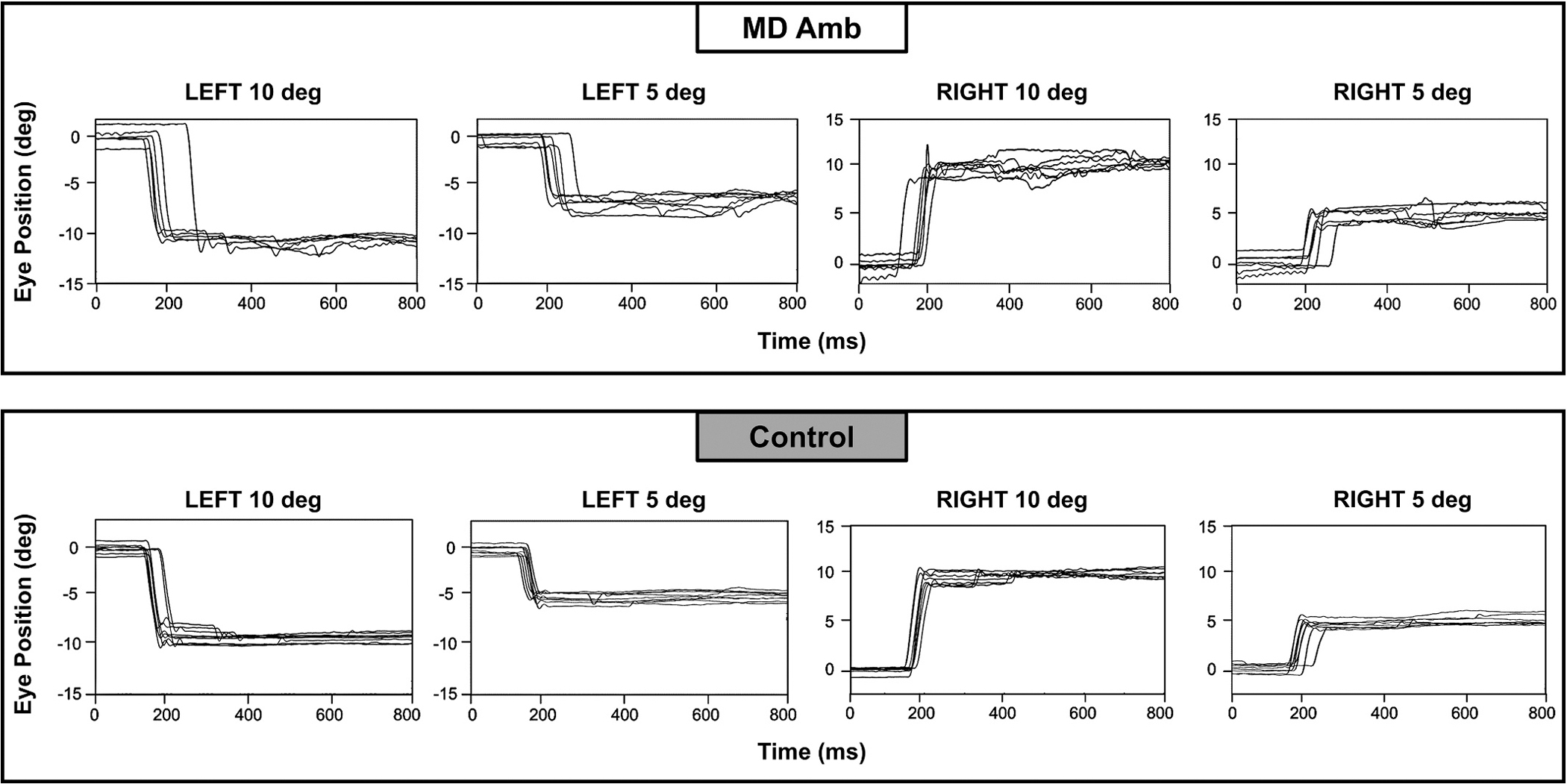
Example saccades showing increased saccade variability (i.e. reduced precision) in a child with MD amblyopia (top) compared to a control child (bottom). Each box shows saccades for trials of the same target position (−10 deg, −5 deg, +5 deg, +10 deg). Each line in the boxes represents one trial.

**Fig. 3.**
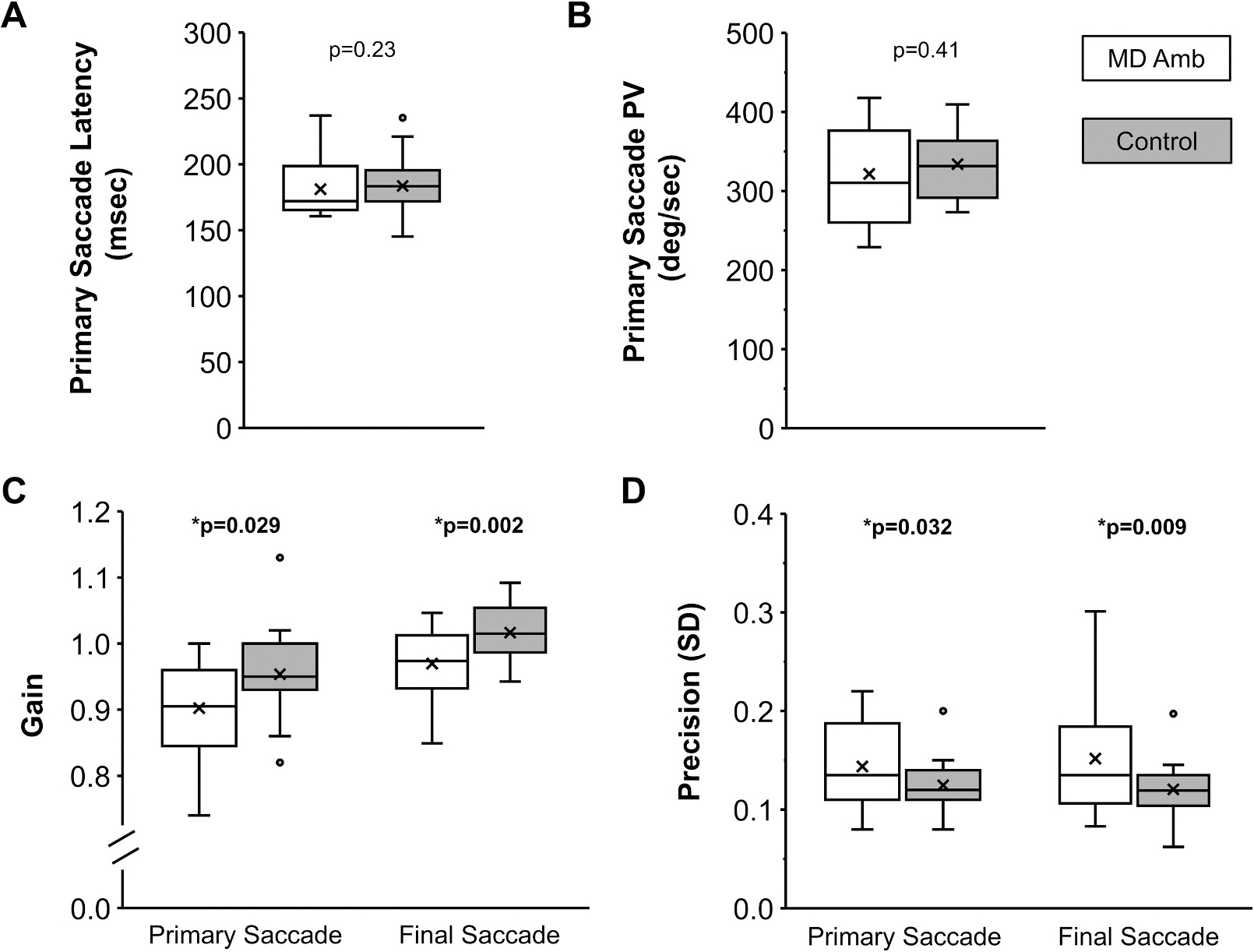
Box and whisker plots showing the distribution of saccade kinematic measures for MD amblyopia (white) compared with controls (grey) for primary saccade latency (A), primary saccade peak velocity; PV (B), primary and final saccade gain (C), and primary and final saccade precision (D). Compared with controls, children with MD amblyopia had similar saccade latency and PV, but had reduced primary and final saccade gain, and higher primary and final saccade variability (i.e. reduced precision). The horizontal line within each box represents the median, the x represents the mean, the boxes correspond to the 25th to 75th percentiles, and the whiskers correspond to the fifth and 95th percentiles. *p is significant.

**Fig. 4.**
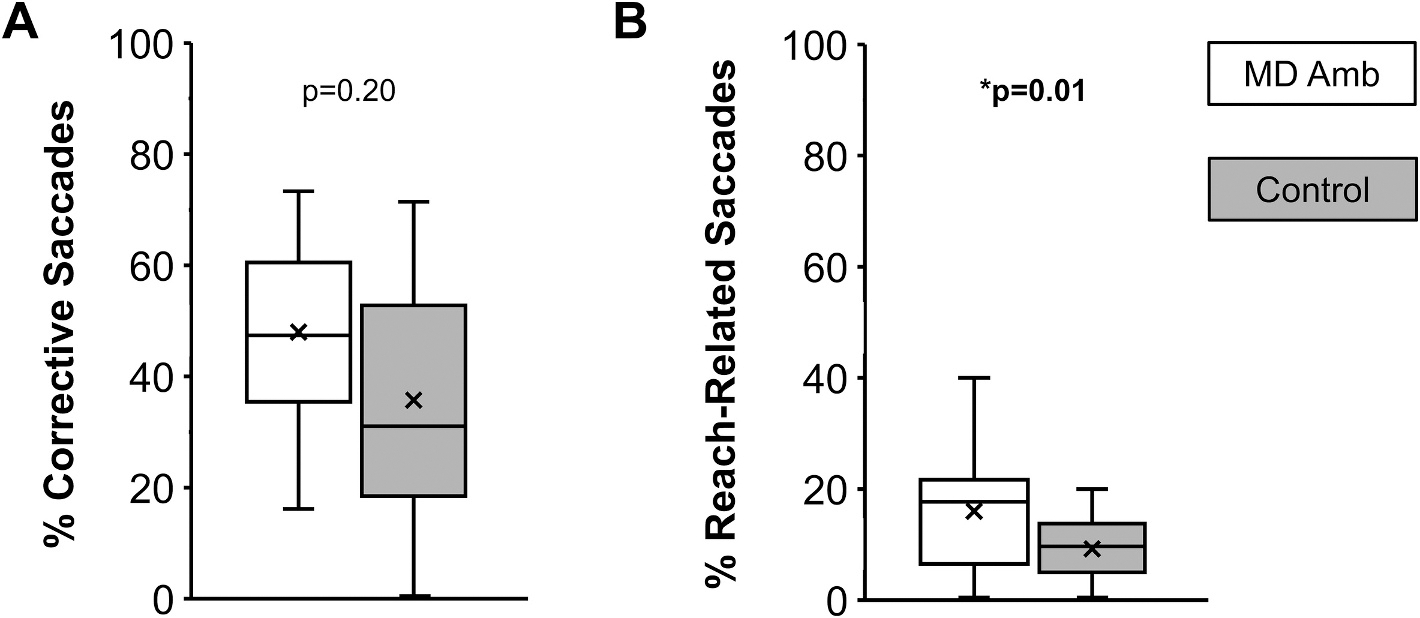
Box and whisker plots showing the distribution of percentage of corrective saccades (A) and reach-related saccades (B) for MD amblyopia (white) compared with controls (grey). Compared with controls, children with MD amblyopia had similar incidence of corrective saccades, but more reach-related saccades. The horizontal line within each box represents the median, the x represents the mean, the boxes correspond to the 25th to 75th percentiles, and the whiskers correspond to the fifth and 95th percentiles. *p is significant.

Only data from the fellow eye (left eye for controls) was analyzed because of poor tracking of the affected eye due to issues from aphakic contact lens, interocular lens, bifocals, and irregular pupils. Saccade kinematic data from 1 child with MD amblyopia and 2 control children were excluded due to having < 14 useable saccade trials because of artifacts, blinks, or poor calibration. See Fig. 2 for example saccades from a typical child with MD amblyopia and a control, and Figs. 3 and 4 for group comparisons of saccade kinematic measures.

There was no group difference for primary saccade latency (control, 173 ± 23 msec vs. MD, 181 ± 22 msec; t_53_ = 1.28, p = 0.23, d = 0.36; Fig. 3A) or peak velocity (control, 334 ± 51 deg/sec vs. MD, 322 ± 61 deg/sec; t_53_ = 0.83, p = 0.41, d = 0.25; Fig. 3B). Compared to controls, the MD amblyopia group had lower primary saccade gain (control, 0.95 ± 0.06 vs. MD, 0.90 ± 0.07; t_53_ = 2.23, p = 0.029, d = 0.67) and lower final saccade gain (1.00 ± 0.05 vs. 0.96 ± 0.05; t_52_ = 3.31, p = 0.002, d = 1.0; Fig. 3C). Compared to controls, the MD amblyopia group had a 17 % reduction in primary saccade precision i.e., higher variability (control, 0.12 ± 0.03 vs. MD, 0.14 ± 0.05; t_53_ = 2.21, p = 0.032, d = 0.66) and a 36 % reduction in final saccade precision (control, 0.11 ± 0.04 vs. MD, 0.15 ± 0.06; t_53_ = 2.75, p = 0.009, d = 0.80; Fig. 3D). While the incidence of corrective saccades was no different than controls (control, 41 ± 19 % vs. MD, 48 ± 16 %; t_53_ = 1.29, p = 0.20, d = 0.38; Fig. 4A), the MD group exhibited a higher percentage of trials with a reach-related saccade (control, 7 ± 6 % vs. MD, 16 ± 11 %; t_53_ = 2.87, p = 0.010, d = 1.10; Fig. 4B).

### 3.2. Reach kinematics

**Fig. 5.**
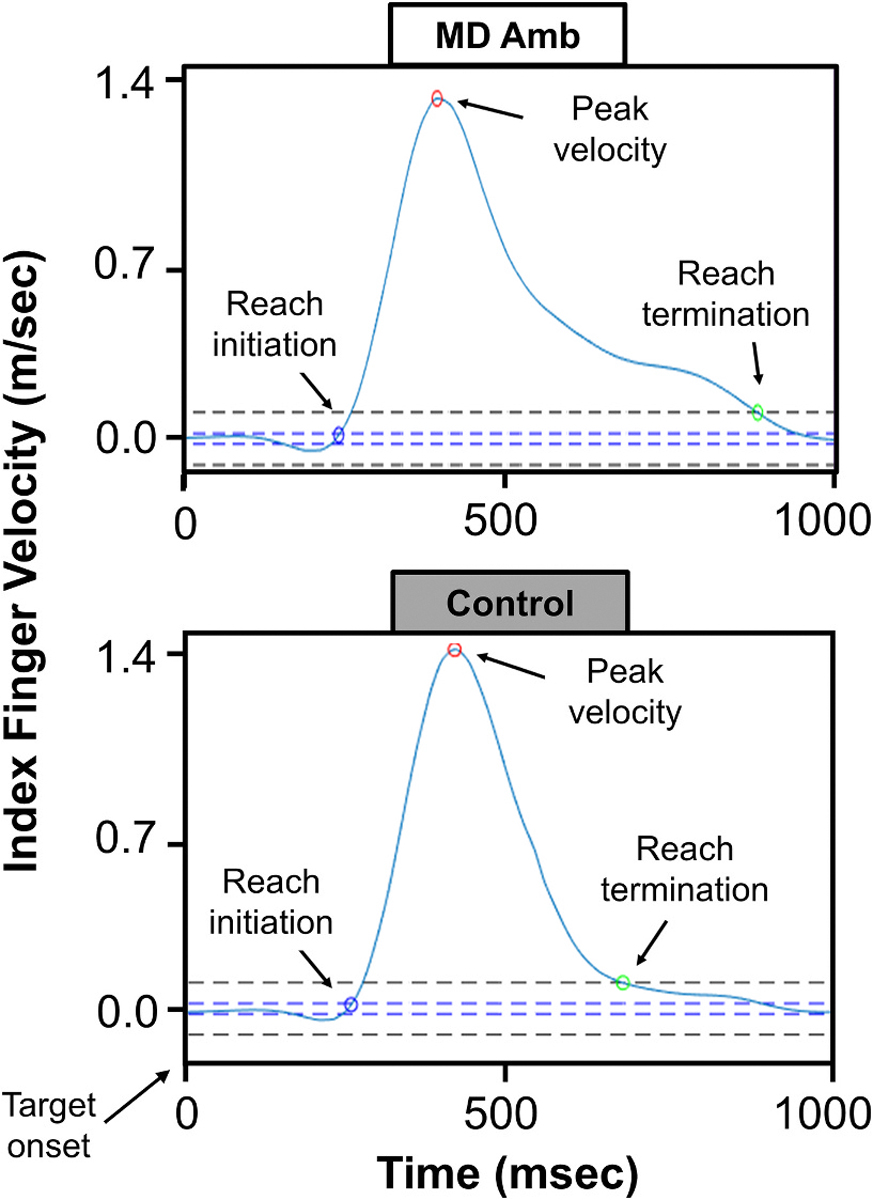
Example index finger movements for one visually-guided reaching trial showing longer total reach duration due to more time spent in the final approach (deceleration phase) for a child with MD amblyopia (top) compared to a control child (bottom). The dashed lines show the thresholds for reach start (blue marker) and reach end (green marker). The red marker shows the peak velocity of the reach. (For interpretation of the references to colour in this figure legend, the reader is referred to the web version of this article.)

**Fig. 6.**
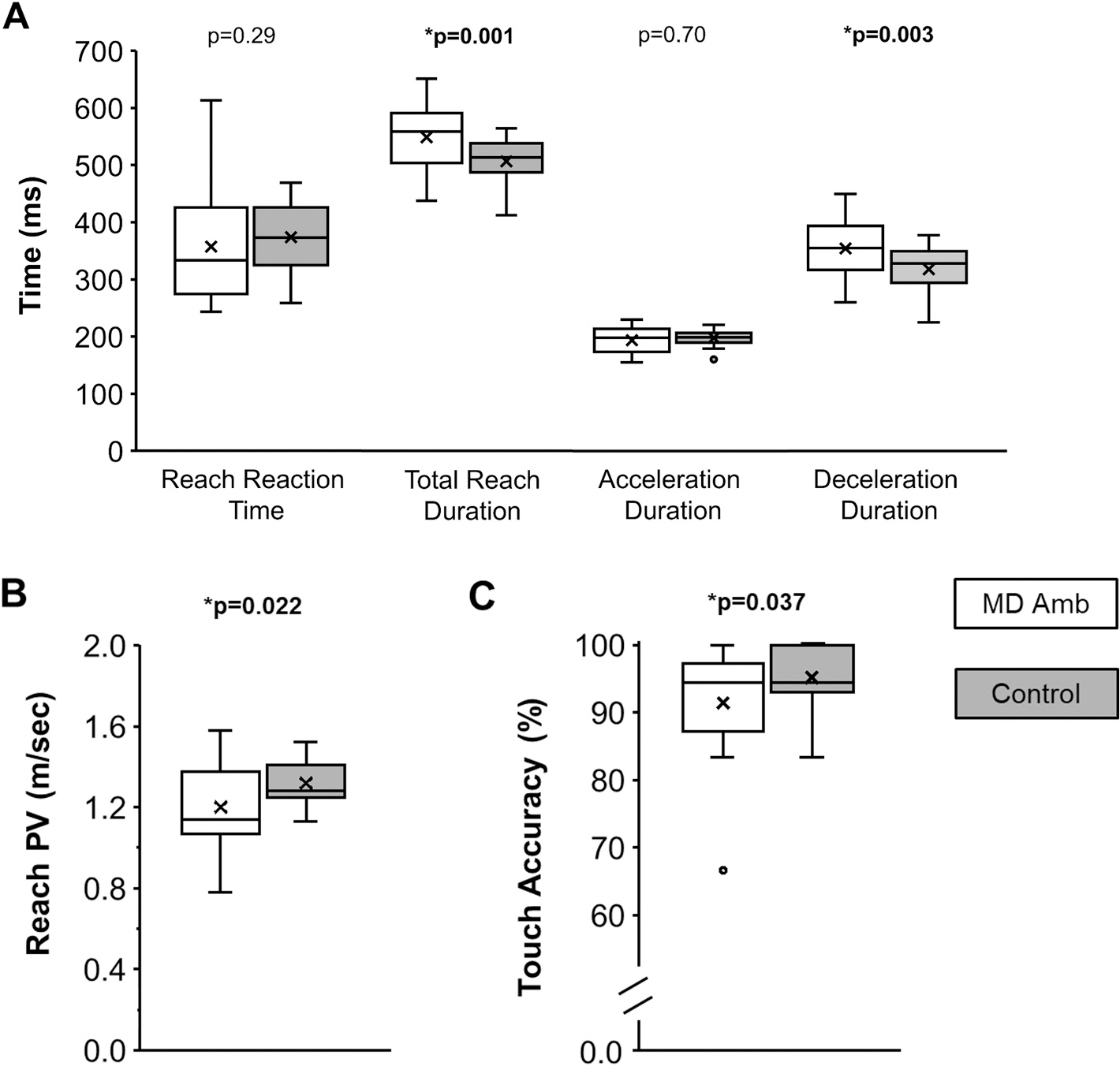
Box and whisker plots showing the distribution of reach kinematic measures for reach reaction time, total reach duration, acceleration duration, and deceleration duration (A), reach peak velocity; PV (B), and touch accuracy (C) for MD amblyopia (white) compared with controls (grey). Compared with controls, children with MD amblyopia had a longer total reach duration due to spending more time in the deceleration phase, slower reach peak velocity, and reduced touch accuracy. The horizontal line within each box represents the median, the x represents the mean, the boxes correspond to the 25th to 75th percentiles, and the whiskers correspond to the fifth and 95th percentiles. *p is significant.

Reach kinematic data were excluded from further analysis for children with < 14 useable trials (4 control, 1 MD amblyopia) to ensure that a minimum of 7 useable trials for each of the left and right dot locations were available for analysis. Compared to controls, the MD amblyopia group were no different on reach reaction time (control, 332 ± 68 msec vs. MD, 357 ± 103 msec; t_55_ = 1.08, p = 0.29, d = 0.31), but had longer total reach duration (control, 503 ± 42 msec vs. MD, 549 ± 59 msec; t_56_ = 3.35, p = 0.001, d = 0.97). There was no difference between groups for acceleration duration (control, 190 ± 21 msec vs. MD, 193 ± 24 msec; t_56_ = 0.39, p = 0.70, d = 0.11). Compared to controls, the MD amblyopia group had longer deceleration duration (control, 312 ± 42 msec vs. MD, 352 ± 52 msec; t_56_ = 3.10, p = 0.003, d = 0.89), lower peak velocity (control, 1.32 ± 0.15 m/sec vs. MD, 1.20 ± 0.22 m/sec; t_56_ = 2.35, p = 0.022, d = 0.68), and lower touch accuracy (control, 96 ± 4 % vs. MD, 91 ± 8 %; U = 228, p = 0.037). See Fig. 5 for individual example trials and Fig. 6 for group means.

### 3.3. Temporal eye-hand coordination

There was no group difference for the saccade-to-reach planning interval (control, 124 ± 65 msec vs MD amblyopia, 116 ± 76 msec; t_653_ = 0.39, p = 0.70, d = 0.12) or the saccade-to-reach peak velocity interval (control, 315 ± 78 msec vs. MD, 305 ± 77 msec; t_53_ = 0.44, p = 066, d = 0.13).

### 3.4. Factors associated with impaired visually-guided reaching

We further probed our data by evaluating clinical and sensory factors associated with impaired reach and saccade kinematics in children with MD amblyopia. Compared with children with an infantile onset (n = 9), children with a congenital onset (n = 8) had lower reach peak velocity (infantile, 1.3 ± 0.2 m/sec vs congenital, 1.1 ± 0.2 m/sec; U = 13, p = 0.021, d = 1.3). No differences were found between the 8 children who had prior strabismus surgery and the 9 children who did not have surgery (all ps ≥ 0.15).

**Fig. 7.**
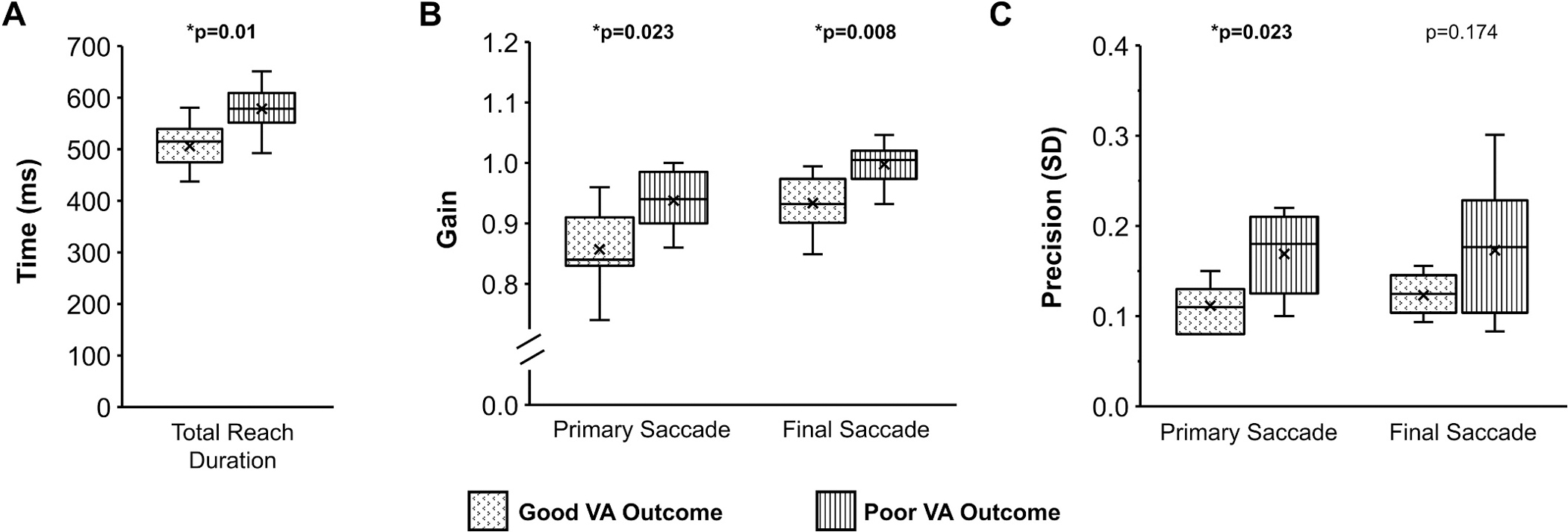
Box and whisker plots showing the distribution of kinematics for total reach duration (A), primary and final saccade gain (B), and primary and final saccade precision (C) for MD amblyopia with a good amblyopic eye visual acuity (VA) outcome (dotted) compared to a poor VA outcome (striped). Compared with a good VA outcome, children with a poor VA outcome had longer total reach duration (A), larger primary and secondary saccade gain (B), but higher primary saccade variability (i.e. reduced precision). The horizontal line within each box represents the median, the x represents the mean, the boxes correspond to the 25th to 75th percentiles, and the whiskers correspond to the fifth and 95th percentiles. *p is significant.

Compared with the 7 children who had a good visual acuity outcome in their amblyopic eye (≤0.6 logMAR), the 10 children with a poor visual acuity outcome (≥0.7 logMAR) had longer total reach duration (poor, 578 ± 47 msec vs good, 506 ± 47 msec, U = 9, p = 0.010, d = 1.6; Fig. 7A), higher primary saccade gain (poor, 0.94 ± 0.05 vs good, 0.86 ± 0.07, U = 10, p = 0.023, d = 1.5; Fig. 7B), and higher final saccade gain (poor, 0.98 ± 0.03 vs good, 0.92 ± 0.05, U = 7, p = 0.008, d = 1.8; Fig. 7B); i.e. primary and final saccade gain in children with a poor visual acuity outcome were similar to that of the control group. Yet, children with a poor visual acuity outcome had more severely reduced primary saccade precision (i.e., higher variability) than children with a good visual acuity outcome (poor, 0.17 ± 0.04 vs good, 0.11 ± 0.03, U = 10, p = 0.023, d = 1.5; Fig. 7C). Further, there was a strong positive correlation of amblyopic eye visual acuity with total reach duration (r_s_ = 0.63, CI_95_ = 0.2–0.9, p = 0.006) and saccade gain (primary, r_s_ = 0.64, CI95 = 0.2–0.9, p = 0.007; final rs = 0.71, CI95 = 0.3–0.9, p = 0.002).

## 4. Discussion

MD amblyopia impacts the development of eye-hand coordination, evidenced by altered saccade and reach kinematics during visually-guided reaching with natural binocular viewing. Reduced gain, coupled with reduced saccade precision by 17–36 % compared to controls, points to frequent undershooting of the target. Thus, reach-related saccades may be needed in children with MD amblyopia to correct positional errors following the primary saccade. Indeed, the MD amblyopia group initiated over twice the amount of reach-related saccades as controls (16 % vs 7 % of trials), consistent with data from strabismic children (16 % vs 8 % of trials) (Kelly et al., 2022). Yet, even with this adjustment, final saccade gain is still low in the MD amblyopia group as a whole and may be the cause of their reduced touch accuracy.

Increased total reach duration in the MD amblyopia group was driven not only by spending more time in the deceleration phase, but also by a decreased reach peak velocity. Despite having more time, children with MD amblyopia were still less accurate than controls (91 % vs. 96 %). More errors and prolonged movement in the final approach are consistent with previous studies of reaching to point in children with strabismus (Kelly et al., 2021; Kelly et al., 2022) and reaching to grasp in children with amblyopia during binocular viewing (Grant et al., 2014; Suttle et al., 2011). Along with slow reaching in the final approach (i.e. deceleration phase), increased reach-related saccades point to a lower quality of or impaired use of visual feedback for on-line motor control (Khan et al., 2006). This is supported by the lower touch accuracy found in the MD amblyopia group. In the MD amblyopia group, most reach-related saccades (71 %) occurred during the deceleration (final) phase of the reach, a period when visual feedback is typically utilized (Khan et al., 2006). It is also plausible that the higher incidence of reach-related saccades reflects fixation instability, which could increase primary saccade variability. Preliminary data from our lab show that the fellow eye of children with MD amblyopia is indeed more unstable during fixation than controls (Patel et al., AAPOS 2021 meeting). Due to how simple and quick each reaching trial was, the task might not be sensitive enough for picking up whether there would be more than one corrective or reach-related saccade. Nonetheless, we still found a difference in the incidence of these eye movements between the groups.

Our findings suggest that binocularly discordant experience from MD amblyopia results in an inability to adapt or form an efficient compensatory strategy during development and may be indicative of impaired on-line control as the reach is being executed. While we did not examine the impact of monocular viewing on controls in this study, a few studies have examined the impact of removing binocular information on eye-hand coordination during reach-to grasp tasks in children. Watt et al. (Watt et al., 2003) found that in visually-normal children aged 5–11 years, removing binocular cues (i.e. monocular viewing) had little effect on overall reach kinematics during a reach-to-grasp task. However, for the older 10–11 year olds, viewing monocularly did lengthen the proportion of time spent in the deceleration phase of the reach – the final approach of the movement. This binocular advantage was absent in younger children. This study suggests that while binocular cues may not play a large role in reach kinematics during childhood, older children do begin to rely on these cues for online control to be more precise in the final stages of the reach. Similarly, Suttle et al. (Suttle et al., 2011) found that children relied less on binocular input than adults to guide their movements. However, even when viewing monocularly, controls aged 4–8 years had shorter movement times and reach durations and better motor control than children with strabismic and anisometropia amblyopia viewing with their fellow eye, indicating that when binocular information is removed, controls still perform much better than children with amblyopia (Alramis et al., 2016). It is important to note that these studies all examined kinematics for a reach-to-grasp task which is much more complex and may require binocular information compared to our simple reach-to-point task. For example, Almaris et al. (Alramis et al., 2016) found a binocular advantage for complex tasks that require more precision such as grasping and threading a small bead compared to placing pegs into a board.

Unlike our previous report of children with strabismus (Kelly et al., 2022), saccade latency was not impacted in children with MD amblyopia during the reaching task. This difference may be due to either the earlier onset of cataracts (here before 2.5 years of age) or the shorter duration of deprivation. Dense cataracts are easier to notice and due to their severe impact on vision, will be treated immediately with extraction and vigorous occlusion of the fellow eye. This may allow for some typical development of saccade latency despite other known ocular motor dysfunctions such as fixation instability found in MD amblyopia. The saccade-to-reach planning interval and the saccade-to-reach peak velocity interval, both measures of temporal eye-hand coordination, occur prior to the final approach of the reach where children with MD amblyopia are slowest. Thus, the lack of differences in our temporal eye-hand coordination measures is unsurprising and aligns with our prior report in strabismic children (Kelly et al., 2022). This again indicates an inefficient use of visual feedback as a possible cause for slower reaching.

We did not report on the saccade kinematics of the amblyopic eye as it was difficult to track for some children. Thus, an important question remains – does the deprived eye contribute to the deficit in eye-hand coordination found in this group? It could be that the children in this group are effectively monocular, with little or no contribution to eye-hand coordination from the deprived eye. Our data support this – most children with MD amblyopia suppressed their amblyopic eye at near (i.e., suppression extended into the periphery) and none had any measurable stereoacuity. Thus, they may be suppressing the centrally present stimuli of 5° and 10° eccentricity, suggesting they are likely using their unsuppressed fellow eye to guide their reaching movements. However, it is clear from our findings that ocular motor function of the fellow eye is impaired during visually-guided reaching, even for a binocular viewing task where the saccade targets are in a fronto-parallel plane to fixation. Previous research points to both sensory and ocular motor deficits in the fellow eye of amblyopic individuals, including motion perception, reading, fixation instability, and saccades (Meier and Giaschi, 2017; Birch et al., 2019; Ho et al., 2005; Kelly et al., 2023; Kelly et al., 2018; Perdziak et al., 2016; Niechwiej-Szwedo et al., 2010). Fellow eye deficits may reflect binocular dysfunction stemming from early discordant experience early in life during a critical period of development. This imbalance of signals can alter binocular neurons in visual areas V2 and V5/MT + that respond to input from either eye (Tao et al., 2014; El-Shamayleh et al., 2010). Evidence from animal models of amblyopia show that neurons in these areas exhibit abnormal neural activity and receptive fields with increased variability (Tao et al., 2014; El-Shamayleh et al., 2010; Wang et al., 2017). Regardless, the hand reaching out to point to the target is in a three-dimensional plane, and children must be visually aware of what the arm/hand is doing to in order to complete the task.

A few clinical factors were related to performance. Those treated for congenital cataracts had lower reach peak velocity compared to those treated for later infantile cataracts. However, reach duration did not differ between these two subsets and no other associations were found, indicating that the type of cataract did not matter much for this study. All cataracts were removed by 2.5 years of age or less, which is likely still well into the critical period of disrupting the development of eye-hand coordination given that adult levels of visuomotor control of upper limbs are not met until adolescence (Niechwiej-Szwedo et al., 2020; Dayanidhi et al., 2013). Compared to a good visual acuity outcome of ≤0.6 logMAR (≤20/80), a poor visual acuity outcome of ≥0.7 logMAR (≥20/100) was associated with slower reaching, but higher saccade gain more similar to that found in the control group. While higher gain in those with a poor visual acuity outcome may seem counterintuitive, it may reflect the deeper suppression of the amblyopic eye and more reliance on the fellow eye to guide saccades. Alternatively, because of the more severe reduction in saccade precision (i.e., more variability) among children with poor visual acuity, higher gain may be an indirect consequence of more instances of overshooting rather than undershooting the target in this group. Indeed, the poor visual acuity group did have a significantly larger proportion of trials with an overshoot compared to the good visual acuity group (35 ± 9 % vs 14 ± 6 %, p < 0.001). Given that the poor VA group had final saccade gain similar to controls, the reduction in gain for the MD amblyopia group as a whole is likely being driven by the good VA outcome group. However, the sample size for these two groups (7, 10) may be too small to make any definitive conclusions.

In our previous study of strabismus (Kelly et al., 2021; Kelly et al., 2022), those who had surgery were slower at reaching and had reduced saccade precision than those who did not have surgery. We postulated that rather than the surgery itself causing deficits, the poorer binocular outcomes may be attributable to the type and severity of strabismus that requires surgery. Our current finding of no difference between children with MD amblyopia who had strabismus surgery versus those who did not solidifies this point. In fact, MD amblyopia typically results in worse binocular outcomes than strabismus (Raab, 2012). All children in the MD amblyopia group in our study had no measurable stereoacuity and most (82 %) suppressed their amblyopic eye at near.

Our study had potential limitations. Microsaccades and fixation instability (e.g. square wave jerks or FMNS) may have been misclassified as corrective or reach-related saccades and may have contributed to the reduced gain and precision found in our study. We excluded saccades <0.4° amplitude in both eyes to minimize the risk of including microsaccades, and visually inspected trials to ensure the quick phases of FMNS sawtooth patterns were not included. Further, it may be difficult to differentiate between two consecutive corrective saccades versus a corrective then a reach-related saccade because our reaching task was quick. We could not control for past eye-hand coordination experience or enrollment in recreational activities; however, the reaching task was simple and all children will have had experience reaching. Further, we did not test the control group monocularly to determine the impact on performance. However, making a control monocular (by patching one eye) is not the same as testing a naturally monocular individual (someone with only one functioning eye). A congenitally monocular individual has had their entire visual system develop and adapt without binocular input but a control who is occluded still has a brain that developed with binocular vision, including binocular neurons and cortical connections. Covering one eye removes binocular rivalry or fusion demands, but the visual system is still “expecting” two inputs. A congenitally monocular individual has no such binocular competition – visual processing has reorganized to maximize the use of the single input. They would have learned to adapt their scanning strategies, head movements, and reliance on motion/parallax cues over time. These adaptive strategies may not be sufficient, as evidence by our data. As mentioned above, removing binocular cues (i.e. monocular viewing) has little effect on overall reach kinematics in visually-normal children especially for easier reaching tasks, and monocular controls still perform better than amblyopic individuals using their fellow eye (Grant et al., 2014; Suttle et al., 2011; Watt et al., 2003; Alramis et al., 2016).

## 5. Conclusions

MD amblyopia resulting from a dense unilateral cataract early in life impacts visually-guided reaching in children. Poorer visual acuity outcomes were associated with worse performance. Identifying the factors contributing to eye-hand coordination deficits in MD amblyopia may guide the advancement of more effective screening tools and interventions to prevent or address motor impairments.

## Figures and Tables

**Table 1:** Mean outcome measures for saccades kinematics, reach kinematics, and temporal eye-hand coordination.

	Outcome	Description

SaccadeKinematics	*Primary Saccade Latency* *Primary Saccade Peak Velocity* *Primary Saccade Gain* *Primary Saccade Precision* *Final Saccade Gain* * *Final Saccade Precision* *Frequency of Corrective Saccades* *Frequency of Reach-Related Saccades*	Time (ms) from target onset to saccade initiation Maximum eye velocity (deg/sec) attained during saccade Ratio of saccade amplitude to target amplitude, measure of accuracy Variability [i.e., standard deviation (SD)] of primary saccade gain] Ratio of final saccade amplitude to target amplitude Variability [i.e., standard deviation (SD)] of final saccade gain] Percentage (%) of trials that included a corrective saccade Percentage (%) of trials that included a reach-related saccade
ReachKinematics	*Reach Reaction Time* *Total Reach Duration* *Acceleration Duration* *Deceleration Duration* *Reach Peak Velocity* *Touch Accuracy*	Time (ms) from target onset to reach initiation Time (ms) from reach initiation to reach termination Time (ms) from reach initiation to peak velocity Time (ms) from peak velocity to reach end Maximum index finger velocity (m/sec) attained during the reach Percentage (%) of trials where the child’s finger covered the dot
Temporal Eye-HandCoordination	*Saccade-to-Reach Planning Interval* *Saccade-to-Reach Peak Velocity Interval*	Time (ms) from primary saccade end to reach initiation Time (ms) from primary saccade end to peak velocity

* Final saccade gain is the total gain of the primary, corrective, and reach-related saccade.

**Table 2:** Group characteristics.

	Deprivation Amblyopia (n = 17)	Control (n = 41)

Sex: F, n (%)	11 (65)	20 (49)
Age, Mean ± SD^a^ years (range)	10.4 ± 2.9 (7.1 to 15.0)	10.4 ± 2.4 (7.0 to 15.7)
Arm Length, Mean ± SD cm (range)	58 ± 7 (50 to 72)	60 ± 7 (51 to 77)
Affected Eye Visual Acuity^b,^ Mean ± SD logMAR (Snellen equivalent) (range)	0.8 ± 0.5 (20/125 ± 5 lines) (0.0 to 1.9)	0.0 ± 0.1 (20/20 ± 1 line) (−0.2 to 0.1)
Good visual outcome (≤0.6 logMAR), n (%)	7 (41)	N/A
Poor visual outcome (≥0.7 logMAR), n (%)	10 (59)	N/A
Fellow Eye Visual Acuity^c^, Mean ± SD logMAR (Snellen equivalent) (range)	0.0 ± 0.1 (20/20 ± 1 line) (−0.1 to 0.1)	0.0 ± 0.1 (20/20 ± 1 line) (−0.2 to 0.1)
Stereoacuity, n (%)		
Present	0 (0)	41 (100)
Not Present	17 (100)	0 (0)
Worth 4-dot Fusion at Near, n (%)		
Fusion	3 (18)	41 (100)
Suppression	14 (82)	0 (0)
Cataract Type, n (%)		
Congenital	8 (47 %)	N/A
Age at Cataract Extraction, Mean ± SD mos (range)	1.5 ± 1.7 (0.3–5.6)^d^	
Affected Eye Visual Acuity, Mean ± SD logMAR	0.8 ± 0.5	
Infantile	9 (53 %)	N/A
Age at Cataract Extraction, Mean ± SD mos (range)	16.1 ± 6.3 (8.7–27.9)	
Affected Eye Visual Acuity, Mean ± SD logMAR	0.9 ± 0.6	
Previous Strabismus Surgery, n (%)		
*Yes*	8 (47)	N/A
*No*	9 (53)	

a SD, standard deviation.

b OD for controls.

c OS for controls.

d 7/8 children had congenital cataract extraction by 1.6 months. One child had a dense cataract first noted by primary care physician at 4 months, with a nuclear cataract and mild microphthalmia diagnosed by a pediatric ophthalmologist at 5 months and extraction at 5.6 months.
